# One more step toward an Asian hypertension guideline

**DOI:** 10.1111/jch.14219

**Published:** 2021-02-16

**Authors:** Ji‐Guang Wang

**Affiliations:** ^1^ Department of Hypertension Ruijin Hospital Centre for Epidemiological Studies and Clinical Trials Shanghai Key Laboratory of Hypertension The Shanghai Institute of Hypertension Shanghai Jiaotong University School of Medicine Shanghai China

## 1 INTRODUCTION

As a council member of the Hypertension Cardiovascular Outcome Prevention, Evidence (HOPE) Asia Network, I should gratefully acknowledge Dr Weber and Dr Lackland for their support in publishing the first special Asia issue in 2020 in the *Journal of Clinical Hypertension* (JCH) in collaboration with Dr Kario, the president of the HOPE Asia Network. The latter organization was established in 2018 as a discussion group of hypertension experts from several Asian countries. In fact, the special issue was published in March 2020, after I had taken over the responsibility of the editor in chief of the JCH in the beginning of the year 2020. I believe that the journey has just started and should continue this year and probably in next few years. Great thanks to Dr Kario for his diligent organizational work on a new special Asia issue this year again in March. In line with the mission of the HOPE Asia Network, this and last special issues focused on various major topics in the management of hypertension in Asia, the most populous continent of the world.

## 2 MANAGEMENT OF HYPERTENSION IN ASIAN COUNTRIES

In the 2020 special Asia issue, the Chinese and Japanese hypertension guidelines were discussed with regard to their relevance for the management of hypertension in Asia. China and Japan had more than two decades history of hypertension guidelines with several updates in both countries, recently in 2018^1^ and 2019,^2^ respectively. Home blood pressure monitoring was also discussed quite extensively, including the publication of the Chinese Hypertension League guidelines on home blood pressure monitoring. The Japanese Society of Hypertension guideline has strong recommendations on the use of home blood pressure monitoring in the diagnosis and therapeutic monitoring of hypertension. Home blood pressure monitoring is probably an effective way for Asian countries to substantially improve the awareness, treatment, and control of hypertension in the next few years or decades, especially with the increasing availability of blood pressure measuring devices with automated transmission and digital platforms.

In the 2020 special Asia issue, a series of short reports were published on the basis of the data obtained in the Asia BP@Home study from 11 countries/regions (n = 1443).^3^ In addition, in these short reports, the authors also discussed the current status and specific concerns in the management of hypertension in their country/region. The control of hypertension is improving in all these countries/regions, but differs between these countries/regions, because there is a big country‐to‐country difference in the economic development and the health care system and policies. Home blood pressure monitoring involves patients and might be a pragmatic strategy to improve the management of hypertension in Asian countries/regions.^4^

A consensus summary on the management of hypertension in Asia was published in the special Asia issue 2020. In this consensus document, Kario and coauthors discussed the Asian characteristics of hypertension, role of home, and ambulatory blood pressure monitoring, and various issues on hypertension‐related diseases. The consensus emphasizes “perfect 24‐hour blood pressure control” guided by home or ambulatory blood pressure monitoring in attempt to achieve the goal of “zero cardiovascular event.” The authors believe that the region‐specific consensus should contribute to optimizing individual and population‐based hypertension management strategies in Asia, and the HOPE Asia Network may provide a paradigm of local interpretation, modification, and dissemination of international best practice to benefit specific populations.

## 3 RELEVANCE OF VARIOUS MAJOR TOPICS FOR THE MANAGEMENT OF HYPERTENSION IN ASIA

The new special Asia issue in 2021 not only expanded its volume but also broadened the topics in relation to the management of hypertension. In addition to the continued discussions on blood pressure measurement strategies, such as home, ambulatory, and central blood pressure measurements, the 2021 issue included several key topics in the treatment of hypertension, special populations, and implementation strategies.

Hypertension is largely an aging‐ and lifestyle‐related cardiovascular disorder, as a consequence of vascular or regulatory system failure. Dietary pattern is quite population‐specific. Various dietary interventions are efficacious in the prevention and treatment of hypertension. Other lifestyle modifications can also be important. Asian countries have a broad range of specific non‐pharmacological approaches, such as Tai Chi and Yoga as exercise therapeutics. Antihypertensive drug treatment is the backbone of hypertension management. A major issue is adherence. There are some discussions on this particular topic. Combination therapy especially in a single pill or even polypill can be a strategy to improve treatment adherence. Innovative drugs are necessary. Angiotensin‐receptor and neprilysin inhibitors may be soon available for the management of hypertension in China and several other Asian countries. It is efficacious in controlling blood pressure, especially nocturnal hypertension, which is more prevalent in Asians than other populations.^5^

In Asia, the treatment of hypertension often starts late. Indeed, many patients start antihypertensive treatment after a cardiovascular complication is present, such as stroke or chronic kidney disease. Stroke is more closely related to hypertension in Asians than other populations. The prevalence of chronic kidney disease is high, regardless of ethnicity in multiethnicity countries such as Singapore. Sleep apnea is a common disorder often in an association with hypertension. The concurrence of hypertension and sleep apnea not only increases the risk of cardiovascular complications, but also the complexity of management. Sleep apnea needs device therapy, such as continuous positive airway pressure. The adherence to device therapy is even lower than to drug treatment.

Telemedicine and digital health technology offers some capacity to implement guideline or consensus recommendations and possibilities to improve treatment adherence. The technological platforms are evolving from phone connection to 5G technology‐based instant interaction between patients and health professionals. With the new technological platform, treatment adherence can be monitored and improved with messaging and counseling. Applications are increasingly available for disease management and health promotion. Digital health with wearable devices will play an increasing role in the management of hypertension in Asia and beyond.

## 4 AN ASIAN HYPERTENSION GUIDELINE

The 2021 special Asia issue really moved one more step further toward an Asian hypertension guideline. Asian countries/regions are diverse in many aspects. However, there are still more similarities than diversities and more consensus than controversies. Most importantly, almost all countries/regions in Asia are facing a fast aging population and an increasing burden of common chronic diseases, especially hypertension. An Asian hypertension guideline may be useful in curbing the burden of hypertension in Asia. After discussions and communications, there has been wide consensus in the management of hypertension among Asian experts, which should help improve control of hypertension in all Asian countries. An Asian hypertension guideline is possible.

Now, it is probably high time to prepare for an Asian hypertension guideline. The 2022 scientific meeting of the International Society of Hypertension will be held in Kyoto, Japan, in conjunction with the scientific meetings of the Asian Pacific Society of Hypertension and the Japanese Society of Hypertension. Although the attendees will be from all over the world, there must be a large attendance of the Asian health professionals working in the field of hypertension. There is about a year to go. The 2021 special issue may behave as a starting point of the first Asian hypertension guideline.

## DISCLOSURE

JG Wang has received consulting and lecture fees from Novartis, Omron, Servier, and Takeda.
